# Drug repurposing using high-throughput screening identifies a promising drug combination to treat adrenocortical carcinoma

**DOI:** 10.18632/oncotarget.26091

**Published:** 2018-09-07

**Authors:** Antonio M. Lerario, Gary D. Hammer

**Affiliations:** Endocrine Oncology, Rogel Cancer Center, University of Michigan, Ann Arbor, MI, USA

**Keywords:** adrenocortical carcinoma, targeted therapies, drug repurposing

Adrenocortical carcinoma (ACC) is a rare malignancy with a dismal outcome. Complete surgical resection remains the only therapeutic option that can cure patients. However, up to 70% of patients who are declared “disease-free” after surgery will recur with metastasis [1]. In this setting, systemic therapies including mitotane +/- polychemotherapy provide a limited survival benefit at the expense of high toxicity. Novel systemic therapies for ACC, more efficacious and less toxic, are urgently needed. However, the rarity and the molecular complexity of ACC, in addition to the paucity of *in vitro* and *in vivo* models, impose significant challenges for developing mechanistic, preclinical and clinical studies investigating new compounds or therapeutic strategies.

To overcome these challenges, a Nilubol *et al.* (published in the current issue of Oncotarget) took advantage of a strategy known as drug repurposing and identified a novel combination of molecularly targeted compounds that exhibited synergistic cytotoxic activity against ACC cell lines *in vitro* and *in vivo*. Drug repurposing is particularly interesting for a rare malignancy such as ACC, for which the development of a potential novel treatment would take a long time since patient accrual to a clinical trial is usually slow in this setting. This strategy can potentially shorten the time interval between preclinical testing and FDA approval, given the known toxicity profiles of the compounds being tested. A similar approach led to a phase I clinical trial (NCT01898715; ClinicalTrials.gov) evaluating ATR-101, a SOAT1 inhibitor originally developed as a lipid-lowering drug that exhibited unexpected adrenal toxicity [2]. It was subsequently demonstrated that ATR-101 triggers caspase-dependent apoptosis in a cholesterol-dependent manner in the presence of nanomolar concentrations of the drug [3].

Nilubol *et al.* provide preclinical evidence that the combination of flavopiridol, a pan-CDK inhibitor, and carfilzomib, a second-generation proteasome inhibitor, exhibits synergistic activity against ACC. These results are in line with new findings on the molecular pathogenesis of ACC, recently published as part of The Cancer Genome Atlas (ACC-TCGA) [4]. According to ACC-TCGA, approximately one-third of ACC exhibit somatic alterations in genes that regulate the G1/S checkpoint, including *CDKN2A, CCNE1, CDK6, RB1,* and *TP53.* The presence of these molecular alterations is associated with high-grade disease and a distinct transcriptional profile characterized by cell cycle activation, genomic instability, and worse clinical outcomes. These observations support the use of CDK inhibitors for ACC therapeutics. Other recent studies further support that targeting cell cycle-dependent kinases may be therapeutically efficacious in ACC [5, 6]. Interestingly, Nilubol *et al.* identified synergism between CDK inhibitors and proteasome inhibitors. A similar combination of drugs, flavopiridol and bortezomib, has been successfully tested in hematological malignancies with good tolerability and a 33% response rate [7].

Of note, while these studies by Nilubol *et al.* provide a rationale for testing targeted approaches involving the use of CDK inhibitors as monotherapy or in combination with other agents such as bortezomib in a clinical trial, an important unresolved consideration is patient selection. ACC-TCGA demonstrated that ACC is a heterogeneous disease, and this should be taken into account when proposing targeted approaches rather than adopting a simplified “*one-size-fits-all*” approach. Indeed, the recent clinical trials on IGF1R inhibitors for ACC taught us a hard lesson on this regard [8, 9]. While *IGF2* is overexpressed in ~90% of ACC and preclinical studies using a variety of IGF1R inhibitors were initially promising, clinical studies demonstrated that most advanced ACC patients did not respond to IGF2/IGF1R therapy, and were largely considered a “failure.” Importantly, a subset of patients (~5-10%) exhibited objective, clinically meaningful responses. Since *in vitro* models of ACC are limited and simply cannot capture the molecular heterogeneity inherent to ACC, future trials should include molecular characterization and subgroup analyses to facilitate appropriate interpretation of results, maximizing the opportunity to identify small subsets of patients that might benefit from specific targeted molecular interventions.

## References

[1] Else T (2014). J Clin Endocrinol Metab.

[2] Vernetti LA (1993). Toxicol Appl Pharmacol.

[3] LaPensee CR (2016). Endocrinology.

[4] Zheng S (2016). Cancer Cell.

[5] Fiorentini C (2018). Endocrine.

[6] Hadjadj D (2017). Aging (Albany NY).

[7] Holkova B (2014). Clin Cancer Res.

[8] Lerario AM (2014). Horm Cancer.

[9] Fassnacht M (2015). Lancet Oncol.

